# Evaluation of Functional Abilities in 0–6 Year Olds: An Analysis with the eEarlyCare Computer Application

**DOI:** 10.3390/ijerph17093315

**Published:** 2020-05-09

**Authors:** María Consuelo Sáiz-Manzanares, Raúl Marticorena-Sánchez, Álvar Arnaiz-González

**Affiliations:** 1 Departamento de Ciencias de la Salud, Facultad de Ciencias de la Salud, Universidad de Burgos, Research Group DATAHES, Pº Comendadores s/n, 09001 Burgos, Spain; 2 Departamento de Ingeniería Informática, Escuela Politécnica Superior, Universidad de Burgos, Research Group ADMIRABLE, Escuela Politécnica Superior, Avd. de Cantabria s/n, 09006 Burgos, Spain; rmartico@ubu.es (R.M.-S.); alvarag@ubu.es (Á.A.-G.)

**Keywords:** computer application, machine learning, early care, special needs

## Abstract

The application of Industry 4.0 to the field of Health Sciences facilitates precise diagnosis and therapy determination. In particular, its effectiveness has been proven in the development of personalized therapeutic intervention programs. The objectives of this study were (1) to develop a computer application that allows the recording of the observational assessment of users aged 0–6 years old with impairment in functional areas and (2) to assess the effectiveness of computer application. We worked with a sample of 22 users with different degrees of cognitive disability at ages 0–6. The eEarlyCare computer application was developed with the aim of allowing the recording of the results of an evaluation of functional abilities and the interpretation of the results by a comparison with "normal development". In addition, the Machine Learning techniques of supervised and unsupervised learning were applied. The most relevant functional areas were predicted. Furthermore, three clusters of functional development were found. These did not always correspond to the disability degree. These data were visualized with distance map techniques. The use of computer applications together with Machine Learning techniques was shown to facilitate accurate diagnosis and therapeutic intervention. Future studies will address research in other user cohorts and expand the functionality of their application to personalized therapeutic programs.

## 1. Introduction

Advances in technology within Industry 4.0, especially those related to the use of computer applications, allow the derivation of data to servers and then to software that can be implemented for the technical analysis of data mining. This has generated a revolution in different fields of knowledge. The application of these advances in the field of therapeutic intervention, especially in diagnosis [1] and precision therapy [2], is within this context. Currently, these advances are already being applied in medical fields for the treatment of cancer [3]. To achieve this, supervised and unsupervised learning Data Mining techniques are applied to facilitate prediction, discovery of behavioural patterns, classification, and grouping of users according to different characteristics that are not established a priori. This facilitates the detection of coincidences in assessed groups [4]. These aspects are very important, since they will help professionals with the development of differential diagnoses and the application of personalized therapeutic intervention programs [5].

Specifically, the application of Industry 4.0, from the philosophy of the Internet of Things [6,7], has an important application in contexts where it is necessary to apply observational methods [8], especially in behavioural observations in early childhood (0–6 years old). In such contexts, the professional (paediatrician, psychologist, pedagogue, occupational therapist, special education teacher, physiotherapist, etc.) has to carry out an observational process on the child’s skills or competences, since this process is the key to a good diagnosis and this, in turn, will be the key to the development of a tailored intervention [9,10].

There are few automated tools that facilitate the observational process, which is a challenge for researchers in this field [11]. One of the handicaps of observational analysis is the difficulty and complexity of recording and interpreting the results [12,13]. For this reason, having a computer tool that allows easy recording and automation of the correction is a significant challenge [14]. The use of Learning Analytics techniques will facilitate more accurate detection of a key development area or areas to initiate therapeutic intervention, as well as to make a choice about the starting point of the therapy. All of these factors speed up the evaluation process and make the intervention process more profitable. However, very few studies have incorporated this methodology into the evaluation processes that facilitate the intervention of professionals in the field of therapy applied to users with special needs at an early age (0–6 years) [5,12].

### 1.1. Technology for Recording and Analysing Observation Results

A desktop software application can be used to solve the recording needs for systematic observations concerning the analysis of user skills in natural contexts. Widely used technologies such as JavaFX or .NET can be used for its development [15]. These technologies allow the introduction of data and its processing with few machine requirements. The objective in subsequent phases is its migration to web or mobile platforms. These technologies also allow the generation of charts or reports. These graphics improve the visualization and interactivity of classic tools, such as spreadsheets. Regarding data storage, these applications can save them in a relational database (e.g., SQLite or PostgreSQL) [16], leaving the possibility of storing them in flat files, such as JavaScript Object Notation (JSON) or Comma Separated Values (CSV), facilitating the integration of data with other tools. Furthermore, for security reasons, it is possible to use the functionality of OneDrive, Dropbox, or Google Drive and Google Docs as cloud storage. These platforms provide authentication and private access mechanisms, which ensure the privacy and protection of data and facilitate interaction with developed applications [17].

In addition, an application can be used to extract dissociated data by taking advantage of cloud storage and applications that allow two-way communication between applications. In this way, the user can consult and extract the data for analysis.

All of this development is accompanied by the use of agile methodologies, preferably that developed by Scrum [18], with short iterations and fast delivery of a functional product. The final result provides an interface in real time with functional Learning Analytics on the degree of development of each user in each of the areas of assessment. It also allows inter-user comparison through the development of a general profile or a profile based on skill or competence development areas.

Once the data have been registered from a client-server solution (with a database server shared by the users of the centre where the therapeutic intervention is being performed) or distributed using a directory and shared files in the cloud (synchronising data from local databases to each machine), the results are recorded, processed, and displayed. In addition, this technology facilitates the application of more complex statistical analyses than mere descriptions and the use of supervised (classification and/or regression) and unsupervised (clustering) Machine Learning techniques through the export of files to more powerful statistical packages such as Statistical Package for the Social Sciences (SPSS) [19] or Waikato Environment for Knowledge Analysis (WEKA) [20], as well as the use of software for the visualization of data in which Machine Learning techniques have been applied, such as Orange [21] or Konstanz Information Miner (KNIME) [22]. In particular, classification and prediction techniques have been shown to be very useful for the design of personalized intervention programs.

Furthermore, a purely web access solution can be used. This allows remote access with a browser from any geographical point without the need to install any software by the client. The web application will provide the options described above, being equivalent in functionality and visual appearance to the previous version. This application will be compatible with the use of the most popular web browsers such as Chrome, Firefox, Edge, Explorer, Safari, and Opera.

### 1.2. Functionalities of the Use of Machine Learning Techniques in the Evaluation of Functional Skills

As already indicated, the use of supervised learning techniques, such as predictive techniques, and unsupervised learning techniques, such as clustering, have special relevance in the field of skills and competence assessment tasks [23]. From the beginning, psychometrics aimed to evaluate the cognitive abilities in human beings through different explanatory constructs [24]. In all of them, behavioural observations and assessment of behaviours on different scales have been and are still applied [23]. In this context, the application of Machine Learning techniques is an opportunity to improve the evaluation and interpretation of results [25]. This results in precise conclusions to the evaluation [26]. In the assessment of cognitive and metacognitive skills, these are directly linked to the diagnosis of development and intellectual abilities. The following is an explanation of the Machine Learning techniques most closely related to the study that we are going to tackle.

Machine Learning techniques allow the relationships between input attributes (sometimes called variables or characteristics) and a target attribute (usually called class) to be determined. The relationship being sought is represented in a structure called a model. This is described as hidden data that are made explicit through a prediction of the value of the target attribute when the values of the input attributes are known [27]. Supervised learning is used, among other areas, in the fields of health and engineering sciences in what has been called health engineering [28,29,30,31,32]. A classifier must be able to assign classes to new examples, even when these have not been used in the training process. The nature of the classification is to discriminate between examples, achieving a reliable prediction of the correct categories of new instances [33,34]. In summary, the objective of supervised learning (a task of Machine Learning) is to obtain input and output mapping whose correct values are provided by a supervisor [35]. Among the possible techniques to be used in supervised learning, the Support Vector Machine technique should be highlighted, as it is based on Vapnik’s statistical learning theory [36]. In the simplest case, with only two linearly separable classes, the aim is to find "the best" separation border between the classes. If the classes are not separable, a penalty factor is introduced to minimize the number of misclassified instances. If the separation is non-linear, a non-linear function that transforms the data set can be used so that the linear separation of the transformed set corresponds to non-linear separation of the source set. The kernel trick technique can also be used [37]. Another technique is discriminant analysis, which is based on multivariate analysis and describe whether there are significant differences in x groups of objects where discriminant variables are detected. Another technique is the Nearest Neighbour (k-nn). This is a supervised learning method that serves to estimate a density function F(x/C_i_) of the predictor variables x for each class C_i_. It is a non-parametric classification that uses a regression method (in the classification, it returns the mode of the class of the closest k examples and in regression, the mean is determined) that estimates the probability density function of element x belonging to class C_i_. This is a method of classifying elements based on training through close examples in the space of possible elements. The best values of k can be obtained using optimization [38]. The applied algorithm is the training of a set of vectors in a multidimensional space, where each example has p attributes from q classes for the classification:

$$x_{i}=\left(x_{1i},\;x_{2i,\;\ldots }x_{pi}\;\right)\;\in X$$

Multiple distance measurements can be used; however, the most frequently used is the Euclidean distance.

$$d\left(x_{i},\;x_{j}\;\right)=\sqrt{\overset{p}{\underset{r=1}{\sum }}{\left(x_{ri}-x_{rj}\;\right)}^{2}}$$

Finally, there is the Decision Tree technique, which applies an algorithm that uses a set of tests or questions organized in a hierarchical way to guide the process of class assignment or to calculate the output value [39]. The criterion for the selection of attributes can be entropy or gain of information, among others. Decision trees are very popular in Data Mining and Machine Learning techniques for several reasons: they are quick to construct, interpretable, and unstable (i.e., small changes in the training set will result in very different trees). The latter property has led to their widespread use in the construction of "multi-classifiers" [34,40]. Among other things, the supervised learning techniques described above are the most widely used. Their application will depend on the characteristics of the sample (standardization of the sample) and the objectives of the research.

Likewise, within the techniques of unsupervised learning, clustering techniques stand out. One of the most used is the k-means algorithm where there is an X set and a distance measurement d: X × X → ℝ. The output of the k-means algorithm is a set of centres *C* = {*c*_1_, *c*_2_,...,*c*_*k*_} which implicitly define a set of clusters in which each point belongs to the cluster represented by the nearest centre, *Φ_C_*(*x*) = argminc *d*(*x*, *c*), the aim being to find the set C that minimises

$$\underset{x\epsilon X}{\sum }d{\left(\Phi_{c}\left(x\right),x\right)}^{2}$$

This implies that each point will be assigned to the nearest centre, thus minimizing the square of the distances of the points to the assigned centre.

Furthermore, when using these resources, it is also significant to consider the use of different techniques to achieve optimization of the results, depending on the characteristics of the samples [41].

The choice of the most appropriate technique depends on will depend on the characteristics of the sample (type of distribution, types of variables) and the objectives of the research.

In summary, technological progress, on the one hand, and the use of Data Mining techniques, on the other, are revolutionizing all areas of knowledge. In particular, their application to the field of health sciences, which includes work on the assessment, diagnosis, and intervention of children with difficulties at ages 0–6, will enable professionals to improve both the recording and interpretation of data. This will increase the accuracy of both diagnosis and intervention. However, very few studies have incorporated this methodology into their assessment to facilitate the intervention of professionals in the field of therapy related to this type of user. The results of this type of research will be very useful for professionals working with children at early ages (0–6 years) and those working with older affected users in functional areas [5].

In view of the above, the objectives of this work were (1) to develop a computer application that allows the recording of the observational assessment of users affected in areas of functional development from 0 to 6 years of age and (2) to test the computer application on users with functional ages of 0–6 years.

## 2. Materials and Methods

### 2.1. Participants

The eEarlyCare (eEarlyCare is a computer application that includes a scale (SFA) that analyzes the functional skills (for ages 0–6 years) in the following areas of development: Food Autonomy, Personal Care and Hygiene, Independently Dresses and Undresses, Sphincter Control, Functional Mobility, Communication and Language, Resolution of tasks in Social Contexts, Interactive and Symbolic Play, Daily Routines, Adaptative Behaviour, and Attention. Both tools are explained in Section 2.2.) software application was used with 22 users with different cognitive impairments who were classified according to Diagnostic and Statistical Manual of Mental Disorders, Fifth Edition (DSM-5) criteria [42], specifically, mild intellectual disability, moderate intellectual disability, and severe intellectual disability. Users also had different schooling modalities (Normal Schooling, Combined Schooling, Schooling in a Specific Special Education Centre). The diagnoses, as well as the recommendations for schooling, were carried out by multidisciplinary teams authorized by the competent Educational Administration in Spain, specifically in the Community of Castilla y León. The characteristics of the sample and the descriptive statistics are shown in Table 1.

### 2.2. Instruments

(a) *eEarlyCare software*. This application was implemented following a client-server solution (with a database server shared by the users of the intervention centre) or distributed using a directory and shared files in the cloud (synchronizing data from local databases to each computer). This application implements two roles: the administrator and the user (therapist or special education teacher) [43]. Further specification of the tool can be found in the first objective in the results section. The functionality of eEarlyCare makes it easy to obtain information about individualized profiles of functional development in 11 areas (Food Autonomy, Personal Care and Hygiene, Independently Dresses and Undresses, Sphincter Control, Functional Mobility, Communication and Language, Resolution of tasks in Social Contexts, Interactive and Symbolic Play, Daily Routines, Adaptative Behaviour, and Attention). It also provides information about the functional development of a set of users within a therapeutic intervention classroom, either in care or in special education centres. It allows the detection of similar and different areas of functional development. This aspect is very interesting regarding the design of different therapeutic intervention programs for users with similar levels of functional development. This aspect facilitates the profitability of resources within the same centre, administration, etc. Furthermore, eEarlyCare allows two roles: the administrator and the specialist (therapist or special educational teacher). Using the first role, users can visualize and configure the users of the centre or centres. Using the second role, users can determine the functional development of all users with whom he/she intervenes and are able to make comparisons by functional areas. This fact is relevant since it can give information about the functional areas and skills most affected in each user and about the common and different aspects in the group of users. All of this facilitates the elaboration of personalized intervention programs for each user and for the group.

(b) *Scale for the measurement of functional abilities in children aged 0–6 years old (SFA)* [44]. This scale is implemented in the eEarlyCare software application. This scale consists of 114 items that are distributed in 11 functional development areas (Food Autonomy, Personal Care and Hygiene, Independently Dresses and Undresses, Sphincter Control, Functional Mobility, Communication and Language, Resolution of tasks in Social Contexts, Interactive and Symbolic Play, Daily Routines, Adaptative Behaviour, and Attention) which, in turn, are subdivided into 33 sub-areas and measured on a Likert type scale from 1 to 5. SFA was developed on the basis of the following instruments: “Portage Guide to Early Education” [45], “Scale of psychomotor development of early childhood (Brunet-Lézine-Revised)” [46], “Battelle Developmental Inventory” [47], and “The Pediatric Evaluation of Disability Inventory” [48].

(c) *SQL Server Express*. This is a free tool that can be downloaded from https://www.microsoft.com/en-US/download/details.aspx?id=55994

(d) *SQL Server Management Studio.* This is a free tool that can be downloaded from https://docs.microsoft.com/es-es/sql/ssms/download-sql-server-management-studio-ssms?view=sql-server-2017

### 2.3. Procedure

First, the research project was presented to the Bioethics Committee of the University of Burgos, and approval was obtained for its development (see Section 2.5). Secondly, once this project was approved, it was presented in the 2018–2019 academic year at the VI Call for Proof of Concept: promotion of the valorisation and commercialisation of the results. This call was financed with FEDER funds. Subsequently, the project was chosen and moved on to the development phase of the application. Once the computer application was developed, the tool was tested in special education and diversity centres, with prior written informed consent from the participating families. The participating children had a previous diagnosis made by multidisciplinary teams that, in all cases, followed the diagnostic criteria of the DSM-5 [42]. Likewise, the project is being continued using a new fund obtained in the 2019–2020 academic year from the VII Call for Proof Concept: promotion of the valorisation and commercialisation of the results, also financed with FEDER funds.

### 2.4. Data Analysis

Asymmetry and kurtosis analysis techniques and descriptive statistics were used. Non-parametric supervised Machine Learning techniques were also applied; the Nearest Neighbour technique (k-nn) and unsupervised learning were used, in particular, the clustering technique k-means. In addition, visualization techniques were applied to the obtained results using the software Orange v. 3.23 [21] (university of Ljubljana University of GNU General Public License, Ljubljana, Slovenia) and SPSS v.24 [19] (International Business Machines Corporation(IBM), New York, NY, USA). 

### 2.5. Ethical Approval

At the beginning of the project, approval was obtained from the Bioethics Committee of the University of Burgos (No. IR 09/2020), as well as authorization from the Provincial Director of Education of Burgos (Spain) and the Principal of the Special Education School "Fray Pedro Ponce de León" Burgos (Spain). Written informed consent was, in each case, requested from the parents or, where applicable, the legal guardians of the participating students. Written informed consent was given in accordance with the Declaration of Helsinki.

## 3. Results

### 3.1. Objective 1

With respect to the first objective ("to develop a computer application that allows the recording of the observational assessment of users affected in areas of functional development from 0 to 6 years of age"), the computer application eEarlyCare was developed. This application has the following characteristics:

#### 3.1.1. Technical Features of eEarlyCare

**Figure 1:**
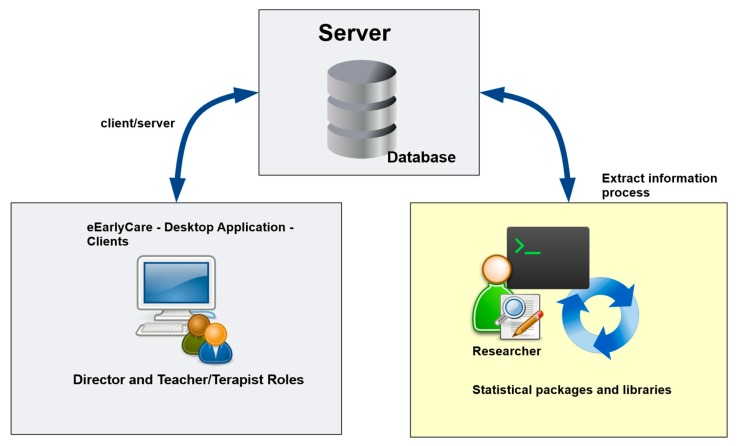
Architecture of the eEarlyCare computer application.

The eEarlyCare computer application was developed following the architecture shown in Figure 1.

**Figure 2:**
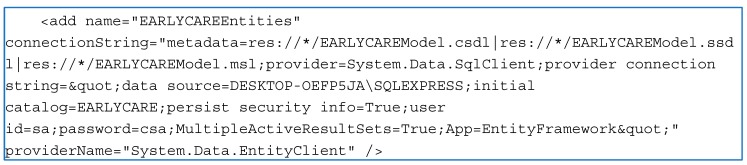
The connection string configured to work with the Structured Query Language (SQL) Server Express installation.

To install the application, the first step is to download the SQL Server Express and install it as the administrator. When everything is installed, the SQL Server Management Studio can be started. It will connect to the instance that was created in the installation. Either the "Windows Authentication" login or the "SQL Server Authentication" login can be used with the superuser created in the installation "sa" + password. Then, the new database is added, and once created, a database copy including tables is restored. Previously, in the directory "C:\Program Files\Microsoft SQL Server\MSSQL14.SQLEXPRESS\MSSQL\Backup", *.bak files with databases to be restored were left. The database to be restored is chosen and accepted. Finally, the program is prepared by decompressing the file and customizing parameters in the eEARLYCare.exe.config. Then, in the connectionStrings section, the connection string is configured to work with the SQL Server Express installation. In the connection string, the instance, the database name, and the user and password must be indicated. An example is shown in Figure 2.

**Figure 3:**
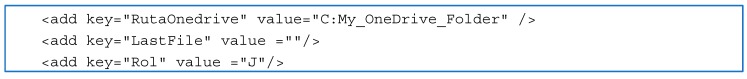
Configuration of administrator role.

Another important step is the configuration of the Administrator role; this role involves the assignment of the registration of therapists. To do this, the following keys are added to the eEARLYCare.exe.config file (see Figure 3).

#### 3.1.2. eEarlyCare Functionality

**Figure 4:**
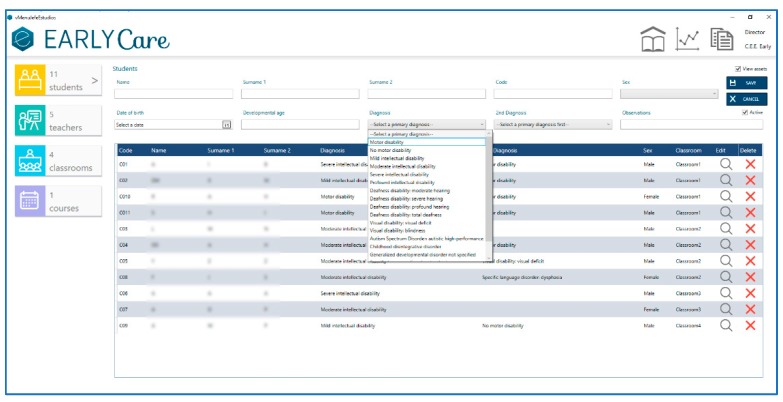
Inserting user data into eEarlyCare.

The first step is to log into the application eEarlyCare, which is accessed through the Administrator role. Figure 4 shows the creation of the users to be observed according to different characteristics (chronological age, sex, developmental age, primary diagnosis, secondary diagnosis, and observations). In addition, each user receives a password to preserve the confidentiality of the data and results.

**Figure 5:**
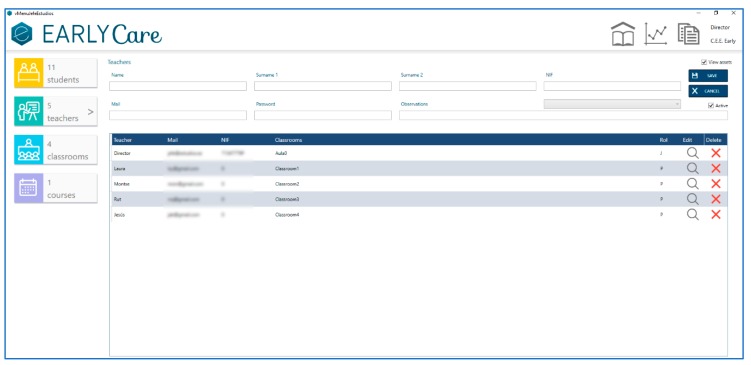
eEarlyCare insertion of therapist/rehabilitator/ special education teacher data.

Then, the Administrator assigns the therapists who will perform the interventions are to each of the users (see Figure 5).

**Figure 6:**
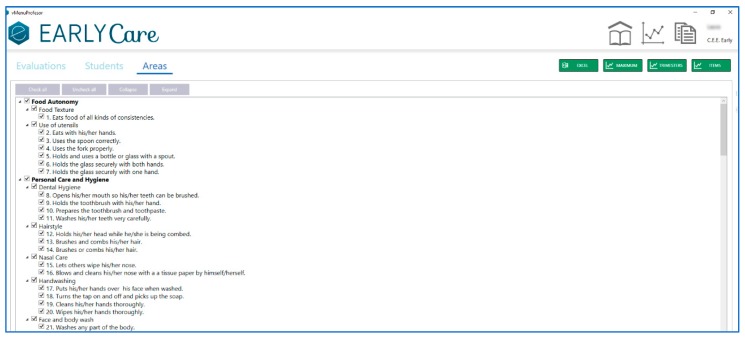
Different dimensions of functional skills assessment in eEarlyCare.

**Figure 7:**
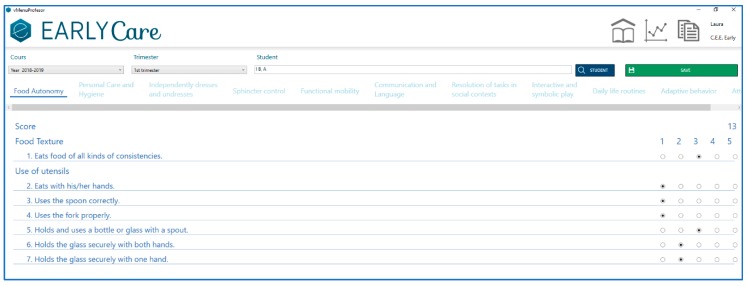
Applying the assessment in eEarlyCare.

Finally, Figure 6 presents the access to the computerized scale. Each therapist fills in the results in three phases: an initial evaluation, a follow-up evaluation, and a final evaluation throughout each academic year in the different functional areas that make up the scale (see Figure 7).

**Figure 8:**
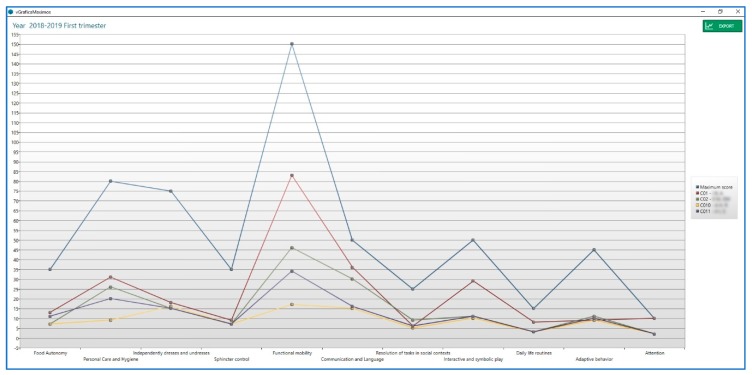
Developmental profiles of different users and comparison with what is expected for their chronological age.

In addition, eEarlyCare allows for Learning Analytics of data for each user and compares expected development based on the chronological age and the difference from the recorded developmental age of each user (see Figure 8).

**Figure 9:**
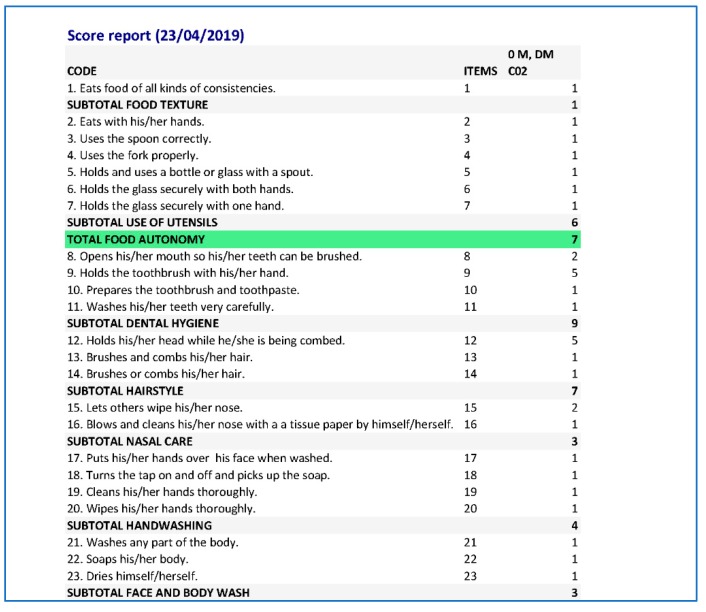
Exporting evaluation data in .xlsx format.

In addition, the eEarlyCare computer application allows the recorded data to be exported in .xlsx format (see Figure 9).

### 3.2. Objective 2

To address the second objective ("to test the computer application on users with functional ages of 0–6 years"), a pilot study was conducted with 22 users with special educational needs, 11 of whom were enrolled in a specific special education centre and 11 of whom were enrolled in ordinary centres and/or in combined schooling modality, that is, part of the time in an ordinary centre and part in a special education centre. The data from the observation made by each therapist were always inserted into the eEarlyCare computer application by the same person in order to neutralize the "type of assessor" effect. The therapist responsible for making the assessment always stayed with the person who inserted the data. Once all the data from the 114 items in the 11 dimensions of the functional skills assessment were recorded, they were exported to an Excel sheet, which, in turn, was imported into the SPSS statistical package v. 24 [19] and Orange data visualization software v. 3.23 [21]. First, in SPSS v.24, the asymmetry and kurtosis indicators showed that the sample distribution was normal. It is important to point out that due to working with this population (children with special educational needs), we were unable to reach the large sample sizes achieved in most studies, and it was necessary to make this type of check in order to adjust the statistical and Data Mining tests to be applied. As can be seen in Table 2, no extreme values of asymmetry or kurtosis were found (the highest value of asymmetry, |2.00|, indicates extreme asymmetry, and kurtosis values between |8| and |20| suggest extreme kurtosis [49]). Since the sample size was less than 30, we used descriptive statistics and non-parametric supervised Machine Learning techniques (the Nearest Neighbour technique, k-nn) as well as unsupervised learning clustering, specifically, k-means. Furthermore, the visualization of the results was obtained by applying Orange software [21].

**Figure 10:**
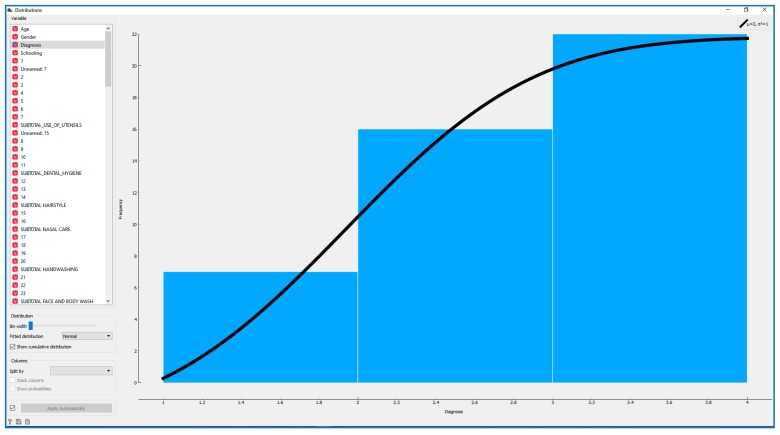
Visualization of the distribution with Orange software.

A visualisation of the distribution was then carried out with Orange (see Figure 10).

Subsequently, the clustering technique, specifically k-means, was applied, and three clusters were found. A cross table between the assignment of users to each cluster and the diagnosis of each one was then constructed (see Table 3).

**Figure 11:**
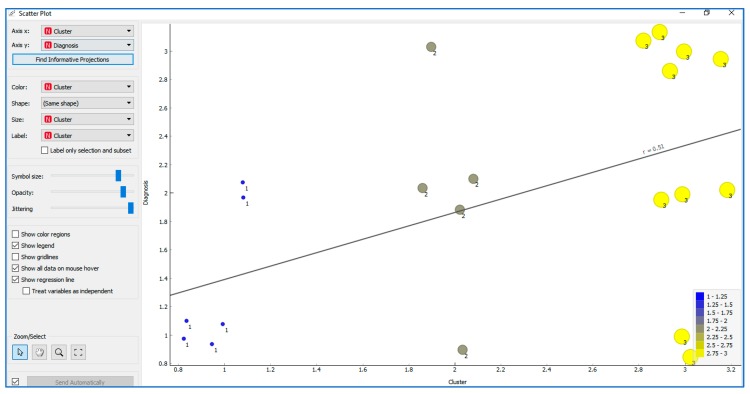
The distribution of users in relation to the degree of disability and their cluster membership, as determined using Orange software.

In Figure 11, the distribution of users among the three clusters can be checked with respect to the diagnostic variable; this was determined using Orange software. The distribution of users with different diagnoses among the three clusters determined from the scores obtained by these users in eEarlyCare is clearly visualised.

**Figure 12:**
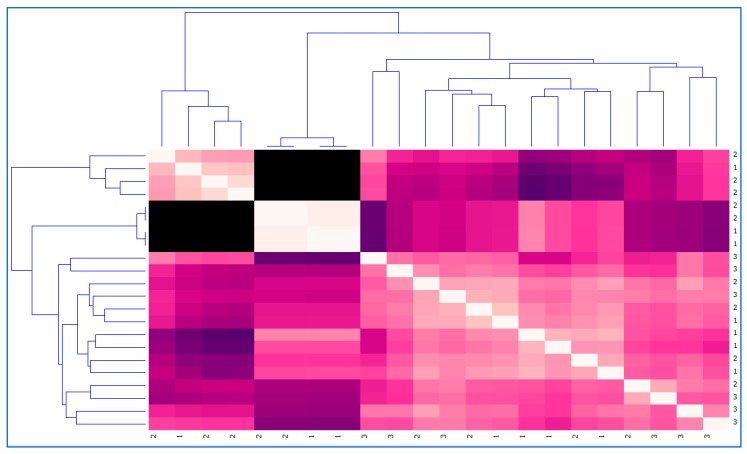
Distance map and dendrogram of the disability degree variable.

Next, in order to check the behaviour of users in the different functional areas and sub-areas analysed by eEarlyCare, a distance map and dendrogram of the disability degree variable was carried out (see Figure 12).

**Figure 13:**
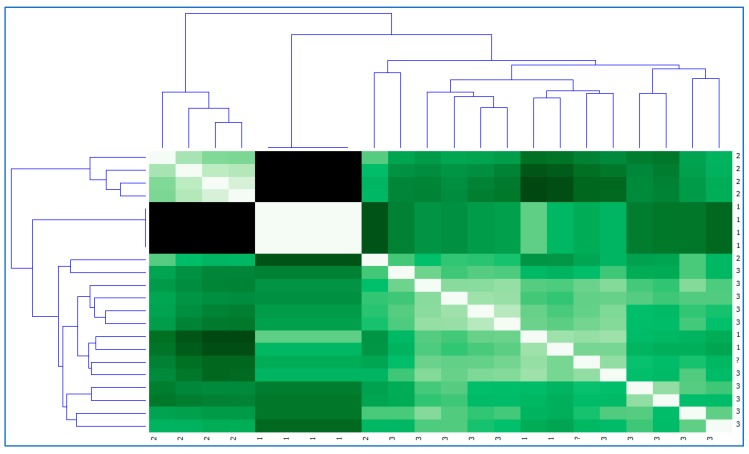
Distance map and dendrogram of the diagnosis for each cluster variable.

Likewise, a distance map and dendrogram of the assignment obtained for each user and their relationship with their behaviours in the different functional areas and sub-areas analysed by the eEarlyCare computer application were constructed (see Figure 13).

As can be seen in Figure 12 and Figure 13, the maps of the distances in relation to assignment to the clusters and grouping in relation to the diagnosis are very similar. However, they are not identical, which corroborates the data found in the cross table (see Table 3).

**Figure 14:**
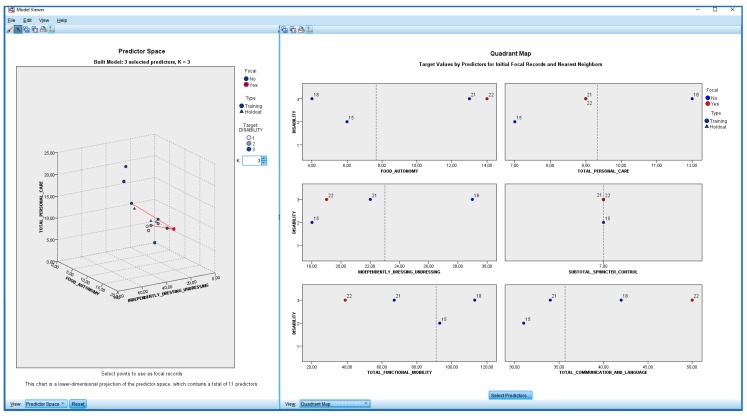
k-nn and distance map in users showing relevant functional areas.

Finally, the Nearest Neighbour (k-nn) supervised learning technique was applied with SPSS v.24 software [19]. We found that the dimensions of eEarlyCare with greatest differences between users were Food Autonomy, Personal Care and Hygiene, and Independently dress and undresses. The map of the quadrant, obtained in SPSS v.24 [19], shows the behaviours in the functional areas of the users in relation to the disability degree variable (see Figure 14).

## 4. Discussion

The use of computer applications that facilitate the recording of observations allows the use of simple Learning Analytics techniques within them. These give professional feedback on the level of development of each user compared to “normal” development standards [6,7,8,9]. In addition, they facilitate the recording of scores in various tabular (.xls, .xlsx, .csv, etc.) formats. The data in these formats can then be exported to databases and servers which, in turn, facilitate the linking of these data with more powerful software in which statistical and Data Mining techniques that will give very important information about the relationship between different variables can be used. For example, the k-nn technique, a supervised learning technique, that guides the prediction, was used in this case. The use of that technique in this study made it possible to determine which variables in the sample had the greatest predictive value. In addition, k-nn made it possible to know which functional skills were most affected. Likewise, k-nn facilitated to detect users with higher risk of functional development. In this study, these variables were Personal Care, Food Autonomy, and Independently dressing and undressing. This is an important milestone for the development of therapeutic intervention programs, since the use of Data Mining techniques will guide the initiation of these programs. Supervised predictive learning techniques determine the area or areas of functional development that are most affected in each case. Similarly, the use of unsupervised learning techniques such as clustering facilitates the identification of specific groupings in the variables studied within a group of users. In this study, the grouping of users according to their behavioural development in each of the functional areas was conducted. It has been proved that other classification criteria, such as the degree of disability, do not allow the grouping of users with the same therapeutic intervention needs. Therefore, clustering techniques can be used to check which of the users have similar needs. Furthermore, the use of distance map and dendrogram techniques will allow the study of the behaviour patterns among participants and the differences between them. In summary, the use of these techniques will allow the elaboration of similar programs as well as the development of these techniques in common spaces and/or schedules, which will enable the intervention to be more precise, sustainable, and effective [1,2,3,4,5]. The eEarlyCare computer application has been shown to be effective for the observational recording of the functional abilities of users with different degrees of cognitive disability [11,12,13,14]. Therefore, the use of computer applications in conjunction with Machine Learning techniques represents a new form of detection and analysis in the field of Health Sciences, supported by technological progress [28,29,30,31,32].

## 5. Conclusions

In this study, an eEarlyCare computer application was developed and was proven to be effective for the recording and simple interpretation of data obtained from the systematic observation in natural contexts of behaviours of children with a developmental age of 0–6 affected by different degrees of cognitive and functional disability. Similarly, eEarlyCare allows the exportation of these data to other computer programs such as SPSS and Orange. It is important to point out that the use of Machine Learning and data visualization techniques guides professional therapists and special education teachers by facilitating precise diagnosis and determination of therapeutic intervention. In addition, it allows the application of statistical techniques and Machine Learning techniques, such as supervised learning, especially predictive, and unsupervised learning, specifically, clustering. These latter techniques are very useful in the fields of Medicine, Psychology, Therapy, and, in general, in the Health Sciences. These techniques allow us to determine the most affected areas and, within them, the distribution of the types of users studied. Similarly, unsupervised automatic learning techniques allow us to determine the groupings between different users using variables that were not previously defined.

In short, the use of properly designed computer applications increases the precision of diagnosis and the personalized adjustment of therapies to the needs of each user at all times. Therefore, this type of computer application together with the use of Machine Learning techniques represents the future for this field, but more studies are needed in this area. However, the research developed in this work obviously has limitations related to the size of the sample and the specific characteristics of the participating users. For this reason, future research, which is already being initiated and financed (see the section on Funding), will focus on extending the application to adults with functional disability and dependency diagnosis. In addition, an extension of the functionality of eEarlyCare is being developed, specifically towards the development of personalized therapies (eEarlyCare Computer Program) which includes the elaboration of personalized and automated programs through the eEarlyCare computer application, starting from the areas and functional behaviours detected as being the most affected. These programs will include specific guidance and also voice assistants that will support the therapist’s systematic work.

## 6. Patents

The computer program eEarlyCare was constructed with the grants received from the Recognized Research Group of the University of Burgos DATAHES and ADAMIRABLE in the VI and VII Editions of the Call for Proofs of Concept: Impulse for the valorisation and marketing of research. The software eEarlyCare computer program has the registration number 00/2019/3855 [43,50].

## Figures and Tables

**Table 1:** Description of the sample with regard to age, gender, disability, and schooling.

Gender	*n*	*M_age_* (months)	*SD_age_*	Rank Age (months)	Disability Degree			Schooling		
					a	b	c	d	e	f
Boys	15	62.40	32.58	24–102.50	6	4	5	2	7	6
Girls	7	72.00	38.18	30–93	1	5	1	2	2	3

Note. *M_age_* = Mean age; *SD_age_* = Standard Deviation age. Degree of disability: a = mild intellectual disability; b = moderate intellectual disability; c = severe intellectual disability; Type of Schooling: d = Normal Schooling; e = Combined Schooling; f = Schooling in a Specific Special Education Center; The children were diagnosed using the DSM-5 criteria. We worked with users who had developmental ages of 0–6 years, although some were chronologically older than 6 years (72 months).

**Table 2:** Analysis of the parameters of asymmetry and kurtosis normality in functional areas (eEarlyCare computer application).

Functional Areas	N	Min	Max	M	SD	S	SES	K	SEK
Food Autonomy	22	0.00	20.00	9.14	6.86	0.29	0.49	–0.98	0.95
Personal Care and Hygiene	22	0.00	25.00	10.68	8.82	0.69	0.49	–0.69	0.95
Subtotal dressing undressing waist upwards	22	0.00	50.00	18.50	16.99	1.05	0.49	–0.08	0.95
Subtotal independently dressing and undressing	22	0.00	75.00	29.18	25.62	0.91	0.49	–0.35	0.95
Sphincter Control	22	0.00	35.00	12.09	12.99	1.23	0.49	–0.21	0.95
Functional Mobility	22	0.00	150.00	73.05	54.20	0.15	0.49	–1.29	0.95
Communication and Language	21	0.00	50.00	26.67	17.27	–0.23	0.50	–0.99	0.97
Resolution of tasks in Social Contexts	22	0.00	21.00	8.59	6.37	0.37	0.49	–0.77	0.95
Interactive and Symbolic Play	22	0.00	46.00	19.14	15.19	0.41	0.49	–1.00	0.95
Daily Routines	22	0.00	15.00	5.50	5.01	0.93	0.49	–0.36	0.95
Adaptive behaviour	22	0.00	11.00	7.50	3.65	–1.70	0.49	1.15	0.95
Attention	22	0.00	10.00	4.14	3.54	0.52	0.49	–1.01	0.95

Note. *M* = Mean; *SD =* Standard Deviation; *Min =* Minimum; *Max =* Maximum; *S =* Skewness; *K* = Kurtosis; *SES =* Standard Error Skewness; *SEK =* Standard Error Kurtosis. Missing values were detected in some functional areas, so they were eliminated.

**Table 3:** Crosstabs between degree of disability and cluster.

Degree of Disability	Ndisability	Cluster 1	%	Cluster 2	%	Cluster 3	%
a	6	4	19.05	1	4.76	2	9.52
b	5	2	9.52	3	14.29	3	14.29
c	10	0	0	1	4.76	5	23.81

Note. Degree of disability: a = mild intellectual disability; b = moderate intellectual disability; c = severe intellectual disability. The children were diagnosed using the DSM5 criteria. Ndisability = Number of users in each type of disability. Missing values were detected and eliminated from the analyses.

## Availability eEarlyCare Computer Application

The eEarlyCare computer application has intellectual property protection no. 00/2019/3855 and was developed within the project “VI Edition of the Call for Proofs of Concept: Impulse for the valorization and marketing of research”. However, the executable application will be available for research purposes to researchers who request it from the corresponding author.
